# Safety and effectiveness of replacement with biosimilar growth hormone in adults with growth hormone deficiency: results from an international, post-marketing surveillance study (PATRO Adults)

**DOI:** 10.1007/s11102-021-01139-2

**Published:** 2021-03-20

**Authors:** Charlotte Höybye, Paolo Beck-Peccoz, Robert D. Murray, Suat Simsek, Günter Stalla, Christian J. Strasburger, Dragan Urosevic, Hichem Zouater, Gudmundur Johannsson

**Affiliations:** 1 Department of Endocrinology and Department of Molecular Medicine and Surgery, Karolinska University Hospital and Karolinska Institute, Stockholm, Sweden; 2 Fondazione Istituto di Ricovero e Cura a Carattere Scientifico Cà Granda Ospedale Maggiore Policlinico, Via Pietro Custodi 16, 20136 Milano, Italy; 3 Leeds Centre for Diabetes & Endocrinology, Leeds Teaching Hospitals NHS Trust, Leeds, LS9 7TF UK; 4 Department of Internal Medicine/Endocrinology, Northwest Clinics Alkmaar, Wilhelminalaan 12, 1815 JD Alkmaar, The Netherlands; 5 Medicover Neuroendokrinologie und Medizinische Klinik und Poliklinik IV der, Ludwig-Maximilians-Universität, Orleansplatz 3, 81667 München, Germany; 6 Department of Medicine for Endocrinology, Diabetes and Nutritional Medicine, Charité Universitätsmedizin, Berlin, Germany; 7 Sandoz Biopharmaceuticals, Fabrikstrasse 2, 4056 Basel, Switzerland; 8 Sandoz Biopharmaceutical, c/o HEXAL AG, Industriestr. 18, 83607 Holzkirchen, Germany; 9 Department of Endocrinology, Sahlgrenska University Hospital, Göteborg, Sweden

**Keywords:** Adults, Biosimilars, Growth hormone, Growth hormone deficiency, Omnitrope^®^

## Abstract

**Purpose:**

To evaluate safety and effectiveness of biosimilar recombinant human growth hormone (rhGH; Omnitrope®) in adults with growth hormone deficiency (GHD), using data from the PATRO Adults study.

**Methods:**

PATRO Adults was a post-marketing surveillance study conducted in hospitals and specialized endocrinology units across Europe. The primary objective was to assess the safety of rhGH in adults treated in routine clinical practice. All adverse events (AEs) were monitored and recorded for the complete duration of Omnitrope® treatment. Effectiveness was evaluated as a secondary objective.

**Results:**

As of January 2020, 1447 patients (50.9% male) had been enrolled from 82 centers in 9 European countries. Most patients had adult-onset GHD (n = 1179; 81.5%); 721 (49.8%) were rhGH-naïve at study entry. Overall, 1056 patients (73.0%) reported adverse events (AEs; n = 5397 events); the majority were mild-to-moderate in intensity. Treatment-related AEs were reported in 117 patients (8.1%; n = 189 events); the most commonly reported (MedDRA preferred terms) were arthralgia (n = 19), myalgia (n = 16), headache (n = 14), and edema peripheral (n = 10). In total, 495 patients (34.2%) had serious AEs (SAEs; n = 1131 events); these were considered treatment-related in 28 patients (1.9%; n = 35 events). Mean (standard deviation) IGF-I SDS increased from – 2.34 (1.47) at baseline to – 0.23 (1.65) at 12 months, and remained relatively stable thereafter (up to 3 years). Body mass index remained stable between baseline and 3 years.

**Conclusion:**

Data from PATRO Adults indicate biosimilar rhGH (Omnitrope^®^) is not associated with any unexpected safety signals, and is effective in adults with GHD treated in real-world clinical practice.

## Introduction

GH deficiency (GHD) in adults is a well-recognized condition amongst adult endocrinologists [1]. Growth hormone (GH) is frequently the first hormone to be affected in hypothalamic-pituitary disorders, however deficiency can remain unrecognized by other specialists, which could lead to delays in referral for treatment. Adults with severe GHD may be eligible for GH replacement therapy, the main goals of which are to reverse the metabolic, functional, and psychological abnormalities associated with adult GHD [2–4]. Treatment of GHD in adults with recombinant human GH (rhGH) has proven to be effective for improving body composition, exercise capacity, skeletal integrity, blood lipid profile, and overall quality of life (QoL) [3, 5–7]. Clinical practice guidelines suggest that the risks associated with rhGH therapy are low [3]. However, extended clinical studies are required to confirm the long-term safety of rhGH therapy in routine clinical practice, particularly with regard to the potential diabetogenic and oncogenic risk.

Omnitrope^®^ (somatropin) is a biosimilar rhGH approved by the European Medicines Agency in 2006, with approval granted on the basis that it matches the reference medicine (Genotropin^®^, Pfizer) in terms of safety, efficacy, and quality. PATRO Adults is an observational, multicenter, longitudinal study of Omnitrope^®^, conducted in hospitals and specialized endocrinology clinics across Europe. The primary objective was to assess the safety of rhGH in adults treated in routine clinical practice. Effectiveness was evaluated as a secondary endpoint [8]. Here, we present safety and effectiveness data from a snapshot analysis carried out in January 2020.

## Methods

The design of the study has been reported previously [8]. Eligible patients were male or female adults who were receiving Omnitrope^®^ treatment and who had provided informed consent. Patients who received treatment with another rhGH medicine before starting Omnitrope^®^ therapy were also eligible for inclusion.

All adverse events (AEs) were monitored and recorded for the complete duration of Omnitrope^®^ treatment. Particular emphasis was placed on: long-term safety; re-occurrence or de novo onset of malignancies; the incidence of hyperglycemia; and the development of glucose intolerance or diabetes. The relationship between AEs and Omnitrope^®^ treatment was independently evaluated by investigator and sponsor assessment, and classified according to the worse case. Laboratory assessments and vital signs were requested to be documented at least once a year, according to routine clinical practice. Effectiveness assessments included analysis of IGF-I levels, anthropometric measures and lipids.

### Data collection/analysis and study populations

Patient data were entered into an electronic case report form (eCRF) at each routine visit. eCRFs were reviewed by data management and on-site monitoring was performed by a contract research organization. Standard descriptive statistics (mean, standard deviation, and frequency) were used to describe continuous parameters (e.g. age, height, weight) and categorical parameters (e.g. gender); median and range were used to describe parameters (e.g. treatment duration) that are not normally distributed.

The safety population consisted of all patients documented within the eCRF at the time of the analysis cut-off date (January 31, 2020). Patients for whom neither a visit date nor a start date of Omnitrope^®^ treatment had been documented were not included. The effectiveness population was a subset of the safety set and consisted of all patients with a documented baseline visit (start of Omnitrope^®^ treatment) and at least one documented visit under Omnitrope^®^ treatment with the corresponding dates.

## Results

### Patient characteristics and diagnostic details

As of January 2020, 1447 patients (50.9% male) had been enrolled from 82 centers in 9 European countries (Czech Republic [n = 9; all rhGH-naïve], France [n = 95; rhGH-naïve, n = 45; pre-treated, n = 50], Germany [n = 388; rhGH-naïve, n = 218; pre-treated, n = 170], Greece [n = 5; all rhGH-naïve], Italy [n = 99; rhGH-naïve, n = 61; pre-treated, n = 38], Netherlands [n = 60; rhGH-naïve, n = 26; pre-treated, n = 34], Spain [n = 48; rhGH-naïve, n = 13; pre-treated, n = 35], Sweden [n = 303; rhGH-naïve, n = 135; pre-treated, n = 168], UK [n = 440; rhGH-naïve, n = 209; pre-treated, n = 231]). At the time of the analysis, 981 patients (67.8%) were active in the study and 466 (32.2%) had discontinued the study. Most patients had adult-onset GHD (n = 1179, 81.5%; childhood-onset, n = 251, 17.3%; missing, n = 17, 1.2%). Also, 721 patients (49.8%) were rhGH-naïve at study entry (adult-onset GHD, n = 669; childhood-onset GHD, n = 42; missing, n = 10) while 726 (50.2%) had been pre-treated with another rhGH.

Further characteristics of enrolled patients are shown in Table 1. Overall, 1267 (99.7%) of patients with combined GHD (n = 1271) had additional pituitary hormone deficiencies (one additional hormone deficiency, n = 228 [17.9%]; 2 additional deficiencies, n = 326 [25.6%]; 3 additional deficiencies, n = 552 [43.4%]; 4 additional deficiencies, n = 161 [12.7%]). Concomitant medication use included thyroid hormones (any; n = 907), hydrocortisone/cortisone (any; n = 799), sex hormones (any; n = 593), and desmopressin (any; n = 73).

**Table 1:** Patient characteristics at enrollment

	Total, n	Gender		GHD onset			Mean (SD) age, years	Mean (SD) BMI, kg/m^2^
		Male, n (%)	Female, n (%)	Childhood, n (%)	Adulthood, n (%)	Missing, n (%)		
Isolated GHD	165	71 (43.0)	94 (57.0)	34 (20.6)	126 (76.4)	5 (3.0)	43.3 (15.2)	29.6 (7.0)
MPHD	1271	659 (51.8)	612 (48.2)	213 (16.8)	1049 (82.5)	9 (0.7)	49.2 (15.5)	29.5 (6.4)
Other	11	7 (63.6)	4 (36.4)	4 (36.4)	4 (36.4)	3 (27.3)	39.6 (15.6)	28.3 (4.4)
Total	1447	737 (50.9)	710 (49.1)	251 (17.3)	1179 (81.5)	17 (1.2)	48.5 (15.6)	29.5 (6.5)

*BMI* body mass index, *GHD* growth hormone deficiency, *MPHD* multiple pituitary hormone deficiency, *SD* standard deviation

### rhGH treatment

The overall mean (SD) baseline Omnitrope^®^ dose was 0.292 (0.238) mg/day (0.276 [0.234] mg/day and 0.309 [0.242] mg/day in males and females, respectively). Among rhGH-naïve patients, the mean (SD) baseline dose was 0.204 (0.14) mg/day; in pre-treated patients, it was 0.379 (0.28) mg/day. At the time of the analysis, the median (range) Omnitrope^®^ treatment duration in the study was 45.3 (0.0–150) months (approx. 3.8 years).

### Treatment discontinuation

As of January 2020, 466 patients had discontinued the study. The most commonly recorded reasons for discontinuation were: ‘other’, n = 150; AE, n = 101; patients not wishing to continue the injections, n = 96; lost to follow-up, n = 49. The most common AEs that resulted in discontinuation (MedDRA preferred term) were headache (n = 7), neoplasm progression (n = 6), prostate cancer (n = 6), arthralgia (n = 5), myalgia (n = 3), pain in extremity (n = 3), edema (n = 3), pneumonia (n = 3), and sepsis (n = 3). AEs of special interest that led to discontinuation (MedDRA system organ class [SOC]) included ‘Neoplasms benign, malignant, and unspecified’ (n = 39) and ‘Metabolism and nutrition disorders’ (n = 4, including diabetes mellitus/type 2 diabetes [n = 3]).

### Adverse events

A summary of reported AEs is shown in Table 2. Overall, 1056 patients (73.0%) reported AEs (n = 5397 events), the majority of which were considered to be mild or moderate in intensity. AEs considered to be related to treatment were reported in 117 patients (8.1%; n = 189 events). The most commonly reported treatment-related AEs (MedDRA preferred terms) were: arthralgia (n = 19), myalgia (n = 16), headache (n = 14), and edema peripheral (n = 10).

**Table 2:** Summary of adverse events

Total number of patients, N = 1447	Patients		AEs, n
	n	%	
Any AE	1056	73.0	5397
Relationship to study drug			
Not suspected	1014	70.1	5205
Suspected	117	8.1	189
Missing/not assessable	3	0.2	2
Intensity			
Mild	858	59.3	3502
Moderate	587	40.6	1414
Severe	171	11.8	302
Missing	66	4.6	179
Changes to rhGH treatment			
Not changed	987	68.2	5016
Increased	28	1.9	38
Reduced	65	4.5	103
Interrupted	62	4.3	90
Permanently discontinued	100	6.9	139
Missing	7	0.5	11
SAEs			
No	969	67.0	4264
Yes	495	34.2	1131
Missing	2	0.1	2
SAE relationship to study drug			
Not suspected	477	33.0	1096
Suspected	28	1.9	35

*AE* adverse event, *rhGH* recombinant human growth hormone, *SAE* serious adverse event

Overall, 39 events of confirmed diabetes mellitus (MedDRA high-level term: Diabetes [including subgroups]) were reported in 37 patients; 32 events (in 30 patients) were newly diagnosed, and 7 events (in 7 patients) were worsening or inadequately controlled diabetes mellitus. Among these 37 patients, 22 (59.5%) had received concomitant statin therapy and 28 (75.7%) had received corticosteroids.

A total of 69 events of malignancies were reported in 60 patients. These events were documented under the SOC ‘Neoplasms benign, malignant, and unspecified’ and classified via medical review as being malignant. Sixty events (in 51 patients) were newly diagnosed; the most common (by MedDRA preferred term) were basal cell carcinoma (13 events in 12 patients), prostate cancer (9 events in 9 patients), malignant melanoma (5 events in 5 patients), and breast cancer (3 events in 3 patients).

In total, 495 patients (34.2%) had SAEs (n = 1131 events); of these, 28 patients (1.9%) had SAEs that were considered to be treatment-related (n = 35 events). The most commonly recorded treatment-related SAEs (MedDRA preferred terms) were diabetes mellitus/type 2 diabetes mellitus (n = 6), lipoma (n = 2), and neoplasm progression (n = 2). Fatal AEs were recorded in 27 patients, the most common being cardiac disorders (n = 10) and cancer (n = 8).

### Effectiveness

**Fig. 1:**
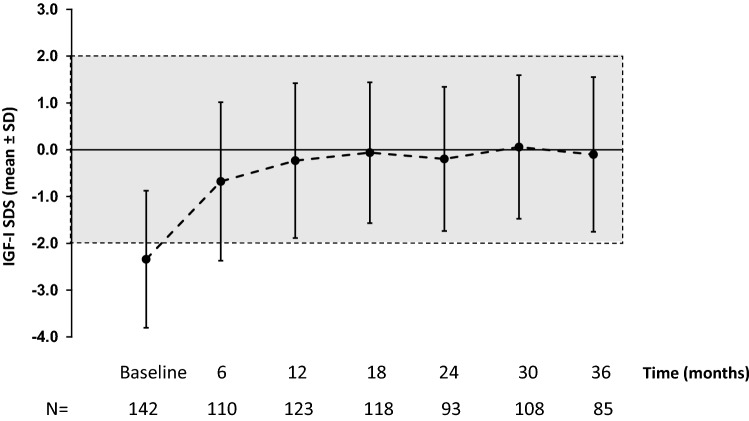
Insulin-like growth factor (IGF)-I SDS over time in rhGH-naïve patients (effectiveness population). The grey shaded area represents the IGF-I target range. *rhGH* recombinant human growth hormone

**Fig. 2:**
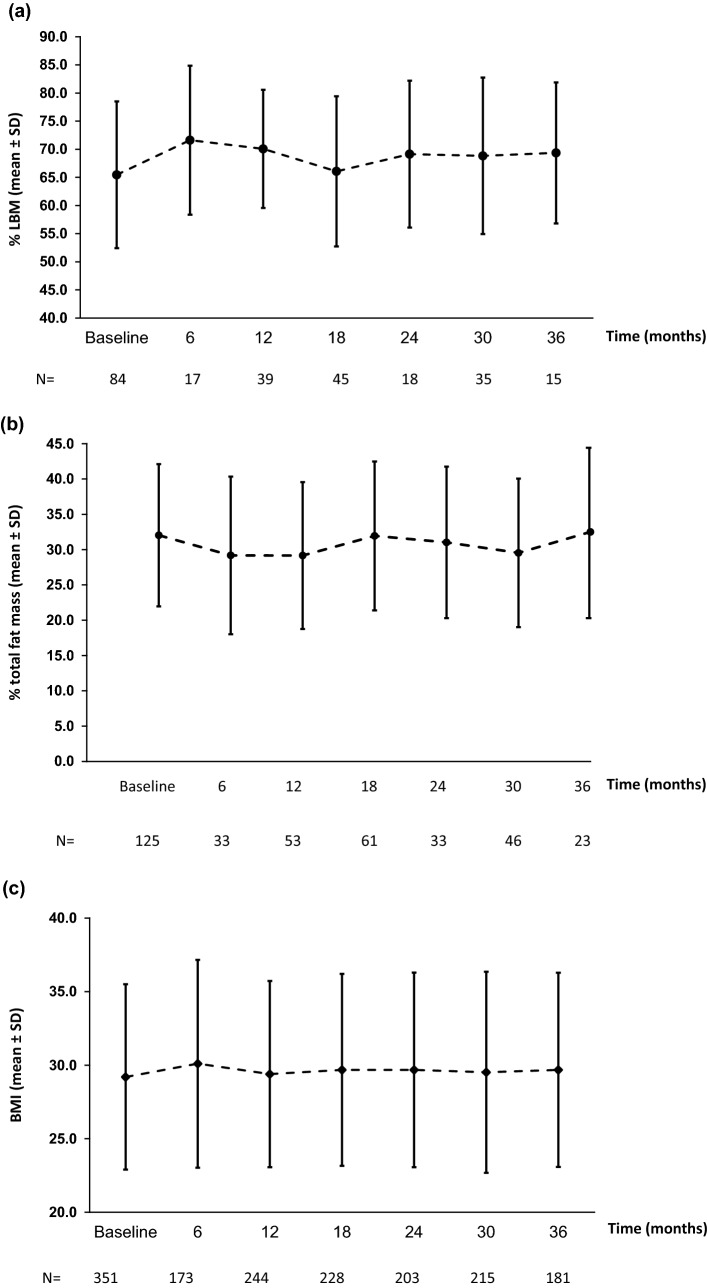
Lean body mass (LBM; **a**), total fat mass (**b**), and body mass index (BMI; **c**) over time in rhGH-naïve patients (effectiveness population). *rhGH* recombinant human growth hormone

Data for effectiveness parameters are presented for patients who were rhGH-naïve on study entry. Mean (SD) IGF-I SDS increased from – 2.34 (1.47) at baseline to – 0.23 (1.65) at 12 months, and remained relatively stable thereafter (up to 36 months; Fig. 1). After 6 months, lean body mass increased by approximately 6% compared with baseline; the increase after 3 years was approximately 4% (Fig. 2a). Total fat mass also improved within the first year of treatment, decreasing by approximately 9% at 12 months, before returning to baseline levels at 36 months (Fig. 2b). BMI remained stable between baseline and 3 years (Fig. 2c).

**Fig. 3:**
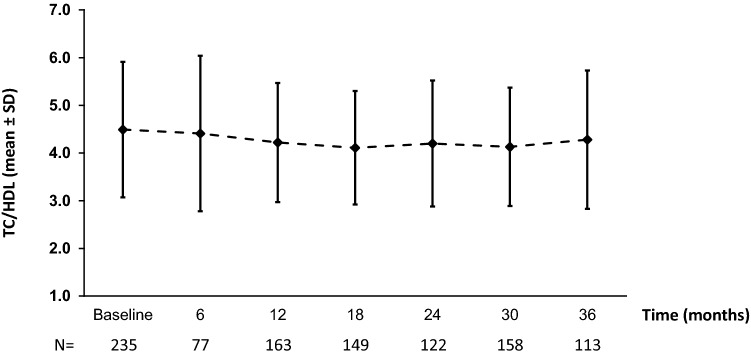
Change in total cholesterol/high-density cholesterol (TC/HDL) over time in rhGH-naïve patients (effectiveness population). rhGH recombinant human growth hormone

Lipid profile improved over time with rhGH treatment. This is exemplified by the decrease in mean (SD) TC/HDL ratio over time, from 4.49 (1.42) at baseline to 4.28 (1.45) after 3 years (Fig. 3).

## Discussion

The PATRO Adults post-marketing surveillance study will provide data on the long-term safety and effectiveness of Omnitrope^®^ treatment in adult patients with GHD in a real-life clinical setting. Based on the current analysis, there have so far been no unexpected safety signals, and treatment is effective (in rhGH-naïve patients).

AEs reported in the current analysis included 32 cases of new-onset diabetes mellitus (in 30 patients) and 7 cases of worsening of pre-existing diabetes mellitus. A previous evaluation of data from PATRO Adults (data cut-off July 2018) examined the impact of rhGH on glucose metabolism and onset of diabetes mellitus [9]. No signals of an increased risk of diabetes mellitus and glucose metabolism disorders were reported, consistent with the experience with other rhGH treatments [10–12]. A review of data from large-scale registry studies of GH replacement therapy suggested that the incidence of diabetes mellitus may only be slightly increased in patients with pre-existing risk factors for diabetes mellitus, and not as a result of rhGH therapy [13].

The current analysis also includes 69 reports of malignancy events (in 60 patients). The occurrence of on-study malignancies in PATRO Adults has been reported previously (based on data from July 2018) [14]. While the data did not in general support a carcinogenic effect of rhGH in adults with GHD, an increased risk of second new malignancies in patients with previous cancer could not be excluded. Again, this is consistent with previous data reported for rhGH medicines in this setting [13, 15].

A number of benefits of rhGH therapy in adult GHD have been previously reported, including improvements in body composition, exercise capacity, skeletal integrity, blood lipid profile, and QoL [3, 16]. The data presented here suggest modest improvements in body composition and blood lipid profile with Omnitrope^®^ treatment (albeit data are included only for patients who were rhGH-naïve on study entry), consistent with previous reports.

It should be highlighted that the PATRO Adults study has several limitations, some of which are common to all observational studies. These include a potential for selection bias due to the inclusion of selected clinics and enrollment of patients from only these clinics. Also, as study data is collected according to routine clinical practice, there is no regular visit schedule, information bias is a possibility (as a result of incorrect or inexact recording), and different techniques may be used to measure the same parameter. Furthermore, there may be under-reporting of AEs because of the extended time periods between patient visits in the study (6–12 months) and restricted consultation time during visits. The relatively small sample size/data sets for some parameters is also a potential limitation and may hinder the interpretation of certain data. Another drawback is the possibility of missing out on useful information due to the set-up of the eCRF. For example, in the present study, the option to select ‘other’ as a reason for study discontinuation resulted in loss of potentially useful data for 150 participants. In the future, avoiding use of general data entry options such as ‘other’ may improve the quality of data collected from such observational studies.

## Conclusion

In summary, data so far from PATRO Adults indicate biosimilar rhGH (Omnitrope^®^) is not associated with any unexpected safety signals, and is effective in adults with GHD treated in real-world clinical practice. However, continued follow-up of adult GHD patients during rhGH therapy and after treatment termination is necessary, to ensure long-term safety.

## Data Availability

The datasets generated during and/or analyzed during the current study are not publicly available as the study is still ongoing, but are available from the corresponding author on reasonable request.
